# Evaluation of Survival Outcomes With Trimodal Therapy as Primary Therapy for Non-organ-confined Bladder Cancer

**DOI:** 10.3389/fonc.2019.01315

**Published:** 2019-12-06

**Authors:** Yadong Guo, Xiaoliang Jie, Aihong Zhang, Wentao Zhang, Ruiliang Wang, Junfeng Zhang, Shiyu Mao, Yuan Wu, Longsheng Wang, Ziwei Zhang, Yang Yan, Ping Wang, Xudong Yao

**Affiliations:** ^1^Shanghai Clinical Medical College, Anhui Medical University, Hefei, China; ^2^Shanghai Tenth People's Hospital, Tongji University, Shanghai, China; ^3^Department of Urology, Shanghai Tenth People's Hospital, Tongji University, Shanghai, China; ^4^Department of Medical Statistics, Tongji University School of Medicine, Shanghai, China

**Keywords:** non-organ-confined bladder cancer, radical cystectomy, trimodal therapy, SEER data, prognosis

## Abstract

**Background:** Currently, the diagnosis of non-organ-confined bladder cancer (NOCBCa) has a very poor prognosis. For patients with NOCBCa, treatments such as radical cystectomy (RC) and systemic chemotherapy have shown survival benefits. However, the relative survival benefits of trimodal therapy (TMT) are unclear.

**Methods:** Patients diagnosed with NOCBCa (cT4bN0M0, cTxN1-3M0, or TxNxM1) were identified in the Surveillance, Epidemiology, and End Results (SEER) database (2004–2015). Patients were grouped based on their definitive treatment for bladder cancer (RC or TMT with maximal transurethral resection, chemotherapy, or radiotherapy). All-cause mortality (ACM) and bladder cancer-specific mortality (BCSM) were assessed by Cox proportional hazard regression and competitive risk models.

**Results:** A total of 2,988 patients met the inclusion criteria and were treated with RC (83.5%) or TMT (16.5%). Patients who underwent TMT had higher 5-year ACM (91.3%) and BCSM (88.8%) results compared to patients who underwent RC (82.6 and 75.0%, respectively) (*P* < 0.001). Adjusted hazard rate (AHR) analysis showed that TMT was associated with higher ACM (AHR: 1.33, 95% CI: 1.15–1.54, *P* < 0.001) and higher BCSM (AHR: 1.32, 95% CI: 1.13–1.54, *P* = 0.001). Subgroup analysis revealed not statistically significant between RC and TMT among patients aged ≥80 years (*P* > 0.05).

**Conclusions:** Compared with TMT, RC is associated with a significant reduction in ACM and BCSM. However, the risks and survival benefits of RC should be weighed, especially in older patients, and our results further suggest that there may be no difference in the prognosis of RC and TMT in patients ≥80 years of age. These results are preliminary and emphasize the need for randomized controlled trials to compare TMT and RC.

## Introduction

It is estimated that 80,470 new cases of bladder cancer (BC) and 17,670 deaths from BC will occur in the United States in 2019 (1). Standard treatment for muscle-invasive bladder cancer (MIBC) is neoadjuvant chemotherapy followed by radical cystectomy (RC) and pelvic lymphadenectomy (LND) (2). A recent report found a 5-year relative survival rate of 77% for all stages combined, and 5-year relative survival rates of 35 and 5% for regional and distant metastatic BCs, respectively (1). Standard treatment for non-organ-confined bladder cancer (NOCBCa) is combination chemotherapy with cisplatin (3). Despite the important survival benefits of this treatment, the life expectancy of NOCBCa remains poor after treatment (4). Although recent advances in immunotherapy have provided hope for cisplatin chemotherapy failure in many NOCBCa patients, the objective response rate is still relatively low (5), and alternative treatments are needed.

Abufaraj et al. recently reported the importance of cytoreductive RC (6), and researchers found that RC-LND improved survival outcomes in patients with NOCBCa (7, 8). Similarly, bladder retention strategies, including trimodal therapy (TMT), have become an effective treatment option in the past few decades. And TMT with largest transurethral resection of bladder tumor (TURBT), chemotherapy, and radiotherapy is another alternative treatment option for patients who are unsuitable or unwilling to receive RC. TMT offers important advantages, maintaining the patient's native bladder and improving quality of life (QoL), leading to increased use of TMT to treat MIBC (9, 10).

A recent large-scale population-based study found no survival benefit for TMT over RC for MIBC (9, 11, 12). However, the survival benefit between RC and TMT for NOCBCa remains unclear. Therefore, based on the Surveillance, Epidemiology, and End Results (SEER) database, we compared survival benefits of RC and TMT to provide alternative treatment for clinicians and patients.

## Methods

### Database and Patient Selections

The SEER database is a population-based cancer registration database that covers ~28% of the US population, recording basic demographics, histology, staging, grading, and treatment for patients with cancer. Using this database, we focused our analysis on patients diagnosed with stage IV (American Cancer Union Committee 6th Edition [AJCC] Cancer Staging Manual) pathologically confirmed primary urothelial cancer between January 1, 2004 and December 31, 2015. We defined patients with stage IV as NOCBCa (cT4bN0M0, cTxN1-3M0, or TxNxM1).

**Figure 1:**
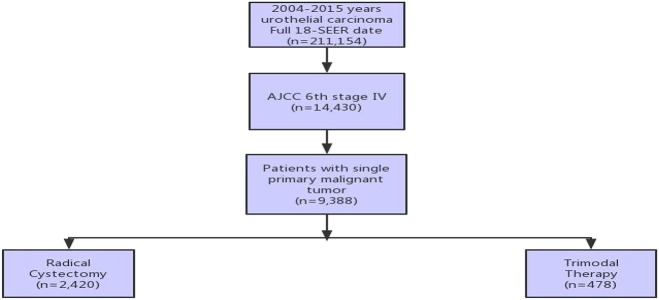
Illustration of patient selection process.

RC was performed according to the program code of the SEER statement. Patients were selected for surgery with or without pelvic LND. The RC group included patients undergoing surgery or receiving radiation therapy or chemotherapy alone. The TMT group included patients who received radiotherapy and chemotherapy after transurethral cystectomy. Lack of survival time and patients who underwent partial cystectomy were excluded. The final cohort included 2,898 patients (Figure 1).

### Statistical Analysis

Propensity score matching is a powerful tool for analyzing observational data because it facilitates comparison of outcomes between similar patient groups. We used propensity score matching to control for selection bias and confounding while comparing prognoses of TMT and RC. The binary logistic regression model was used to estimate propensity scores for each patient. In light of previous research and clinical knowledge, we included age at diagnosis, sex, race, marital status, tumor grade, and derived AJCC T, N, M stages in the propensity score model. We performed 1:2 matching of patients undergoing TMT with patients undergoing RC, based on the nearest-neighbor matching algorithm. Standardized difference was used to assess the covariate balance; an absolute standardized difference <0.10 indicated a balance of covariates across the 2 groups.

A Cox proportional hazards regression model was used to estimate hazard ratios (HRs) of all-cause mortality (ACM) in matched and all patients, respectively. We conducted competing risk analyses to compare bladder-specific cancer mortality (BCSM) between TMT and RC in matched and all patients, respectively. All statistical tests were 2-sided, and all analyses were performed with Stata/MP 14.0 and R packages.

## Results

### Patient Characteristics

We summarized patient demographic data for treatment type before and after adjusting for propensity scores (Table 1). Of the 2,988 patients identified, 478 (16.5%) received TMT and 2,420 (83.5%) underwent RC. Median follow-up time was 13 months [interquartile range (IQR): 7–27]: 15 months (IQR: 8–29) for RC, and 10 months (IQR: 5–17) for TMT. Median age of diagnosis was 66 years (IQR, 58–74): 65 years (IQR, 58–74) for RC, and 67 years (IQR, 59–77) for TMT. A total of 1,888 patients (65.1%) died of BC: 1,522 RC patients (62.9%), and 366 TMT patients (76.6%).

**Table 1:** Patient demographics and clinical characteristics stratified by treatment type, with and without propensity score matching (ratio 2:1).

**Characteristic**	**Unmatched**			**Matched**		
	**TMT**	**RC**	***SD***	**TMT**	**RC**	***SD***
	**(*n* = 478)**	**(*n* = 2,420)**		**(*n* = 478)**	**(*n* = 956)**	
Age (years), *n* (range)	67.5 (59.0–77.0)	65.0 (58.0–74.0)	0.200	67.5 (59.00–77.0)	66.0 (58.0–74.0)	0.178
**Marital status**						
Married	247 (51.7)	1392 (57.5)	−0.118	247 (51.7)	547 (57.2)	−0.112
Divorced/widowed	139 (29.1)	616 (25.5)	0.082	139 (29.1)	242 (25.3)	0.085
Single	76 (15.9)	345 (14.3)	0.046	76 (15.9)	135 (14.1)	0.050
Unknown	16 (3.4)	67 (2.8)	0.034	16 (3.4)	32 (3.4)	0.000
**Sex**, ***n*** **(%)**						
Male	359 (75.1)	1611 (66.6)	0.189	359 (75.1)	684 (71.6)	0.080
Female	119 (24.9)	809 (33.4)	−0.189	119 (24.9)	272 (28.5)	−0.080
**Race**, ***n*** **(%)**						
White	411 (86.0)	2079 (85.9)	0.002	411 (86.0)	806 (84.3)	0.047
Black	44 (9.2)	190 (7.9)	0.049	44 (9.2)	92 (9.6)	−0.014
Other	23 (4.8)	146 (6.0)	−0.054	23 (4.8)	58 (6.1)	−0.056
Unknown	0 (0.0)	5(0.2)	−0.065	0 (0.0)	0 (0.0)	
**Tumor grade**, ***n*** **(%)**						
Grade I to II	16 (3.3)	42 (1.7)	0.103	16 (3.3)	22 (2.3)	0.063
Grade III	142 (29.7)	755 (31.2)	−0.032	142 (29.7)	285 (29.8)	−0.002
Grade IV	282 (59.0)	1543 (63.8)	−0.098	282 (59.0)	604 (63.2)	−0.086
Unknown	38 (8.0)	80 (3.3)	0.202	38 (8.0)	45 (4.7)	0.133
**Derived AJCC T stage**, ***n*** **(%)**						
T1	56 (11.7)	60 (2.5)	0.366	56 (11.7)	41 (4.3)	0.276
T2	246 (51.5)	463 (19.1)	0.719	246 (51.5)	386 (40.4)	0.224
T3	43 (9.0)	1068 (44.1)	−0.867	43 (9.0)	192 (20.1)	−0.318
T4	133 (27.8)	829 (34.3)	−0.140	133 (27.8)	337 (35.3)	−0.160
**Derived AJCC N stage**, ***n*** **(%)**						
N0	199 (41.6)	212 (8.8)	0.818	199 (41.6)	87 (9.1)	0.806
N1	127 (26.6)	1095 (45.3)	−0.397	127 (26.6)	434 (45.4)	−0.400
N2	105 (22.0)	1060 (43.8)	−0.478	105 (22.0)	394 (41.2)	−0.423
N3	11 (2.3)	35 (1.45)	0.063	11 (2.3)	26 (2.7)	−0.027
Nx	36 (7.5)	18 (0.7)	0.346	36 (7.5)	15 (1.6)	0.289
**Derived AJCC M stage**, ***n*** **(%)**						
M0	175 (36.6)	1972 (81.5)	−1.025	175 (36.6)	709 (74.2)	−0.816
M1	298 (62.3)	429 (17.7)	1.023	298 (62.3)	230 (24.1)	0.838
Mx	5 (1.1)	19 (0.8)	0.027	5 (1.1)	17 (1.8)	−0.062

*TMT, trimodal therapy; RC, radical cystectomy; SD, standardized difference*.

### Association of Treatment Options With ACM and BCSM of Patients

**Figure 2:**
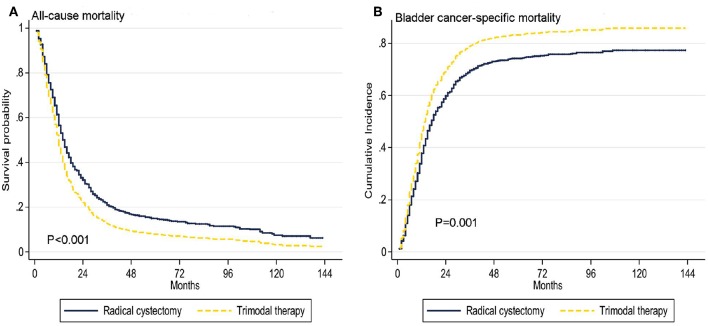
Adjusted survival curves for all-cause mortality **(A)** and bladder cancer-specific mortality **(B)** by radical cystectomy (RC) and trimodal therapy (TMT) treatment options after weighting.

Treatment options were associated with ACM and BCSM of patients. The 1-, 3-, and 5-year ACM of patients were as follows: 41.9, 75, and 82.6% for RC, and 57.2, 87.9, and 91.3% for TMT, respectively. The 1-, 3-, and 5-year BCSM of patients were as follows: 37, 71.5, and 75% for RC, respectively, and 54.2, 85.6, and 88.8% for TMT, respectively (Table 2, Figure 2).

**Table 2:** The 1-, 3-, and 5-year all-cause mortality and bladder cancer-specific mortality of patients after radical cystectomy and trimodal therapy.

**Therapy**	***n* (%)**	**Weighted**		
		**1-year (95%CI)**	**3-year (95%CI)**	**5-year (95%CI)**
**BLADDER CANCER-SPECIFIC MORTALITY**				
RC	2,420 (83.5%)	0.370 (0.329, 0.412)	0.715 (0.670, 0.759)	0.750 (0.705, 0.793)
TMT	478 (16.5%)	0.542 (0.499, 0.586)	0.856 (0.821, 0.887)	0.888 (0.854, 0.917)
**ALL-CAUSE MORTALITY**				
RC	2,420 (83.5%)	0.419 (0.377, 0.463)	0.750 (0.709, 0.790)	0.826 (0.785, 0.863)
TMT	478 (16.5%)	0.572 (0.530, 0.614)	0.879 (0.849, 0.907)	0.913 (0.884, 0.937)

*RC, radical cystectomy; TMT, trimodal therapy*.

Overall, multivariate and propensity matched score adjustment analyses found that the patient's age of diagnosis, T, N, M stage, and treatment were related to prognosis (*P* < 0.05, Table 3). Compared to RC, TMT was associated with higher ACM [adjusted HR (AHR): 1.33, 95% CI: 1.15–1.54, *P* < 0.001, Table 3]. Similarly, in the competitive risk model, there was a difference in BCSM between patients treated with TMT and RC (AHR: 1.32, 95% CI: 1.13–1.54, *P* = 0.001, Table 3).

**Table 3:** Proportional hazards regression model for the all-cause mortality and bladder cancer-specific mortality according to treatment type.

**Covariate**	**Bladder cancer-specific mortality**		**All-cause mortality**	
	**HR (95% CI)**	***P*-value**	**HR (95% CI)**	***P*-value**
**Age at diagnosis (years)**				
≤60	1 (Reference)		1 (Reference)	
61–79	0.96 (0.83–1.11)	0.567	1.15 (0.99–1.32)	0.053
≥80	1.25 (0.99–1.57)	0.057	1.60 (1.30–1.96)	<0.001
**Sex**				
Male	1 (Reference)		1 (Reference)	
Female	1.10 (0.95–1.28)	0.207	1.07 (0.93–1.23)	0.369
**Marital status**				
Married	1 (Reference)		1 (Reference)	
Divorced/ Widowed	1.05 (0.90–1.23)	0.515	1.21 (1.05–1.39)	0.01
Single	1.06 (0.87–1.28)	0.572	1.22 (1.02–1.47)	0.034
Unknown	1.34 (0.95–1.89)	0.096	1.13 (0.81–1.58)	0.468
**Race**				
White	1 (Reference)		1 (Reference)	
Black	1.00 (0.77–1.29)	0.97	1.05 (0.85–1.30)	0.659
Other	0.98 (0.73–1.31)	0.895	0.82 (0.62–1.07)	0.143
**Derived AJCC T stage**				
T1	1 (Reference)		1 (Reference)	
T2	0.53 (0.39–0.73)	0.472	1.09 (0.84–1.41)	0.519
T3	0.69 (0.58–0.80)	<0.001	1.92 (1.45–2.55)	<0.001
T4	0.96 (0.80–1.16)	<0.001	2.10 (1.62–2.73)	<0.001
**Derived AJCC N stage**				
N0	1 (Reference)		1 (Reference)	
N1	0.98 (0.79–1.20)	0.815	1.05 (0.87–1.28)	0.602
N2	1.02 (0.83–1.27)	0.827	1.10 (0.91–1.33)	0.345
N3	1.57 (1.06–2.32)	0.026	1.70 (1.14–2.52)	0.009
Nx	1.28 (0.94–1.73)	0.114	1.18 (0.86–1.63)	0.299
**Derived AJCC M stage**				
M0	1 (Reference)		1 (Reference)	
M1	1.63 (1.40–1.91)	<0.001	1.73 (1.49–2.00)	<0.001
Mx	1.17 (0.74–1.86)	0.498	1.33 (0.85–2.11)	0.216
**Grade**				
Grade I to II	1 (Reference)		1 (Reference)	
Grade III	1.00 (0.66–1.52)	0.984	0.91 (0.63–1.33)	0.632
Grade IV	0.91 (0.61–1.38)	0.67	0.87 (0.60–1.26)	0.469
Unknown	1.04 (0.65–1.65)	0.876	0.93 (0.60–1.44)	0.742
**Treatment group**				
Radical cystectomy	1 (Reference)		1 (Reference)	
Trimodal therapy	1.32 (1.13–1.54)	0.001	1.33 (1.15–1.54)	<0.001

**Figure 3:**
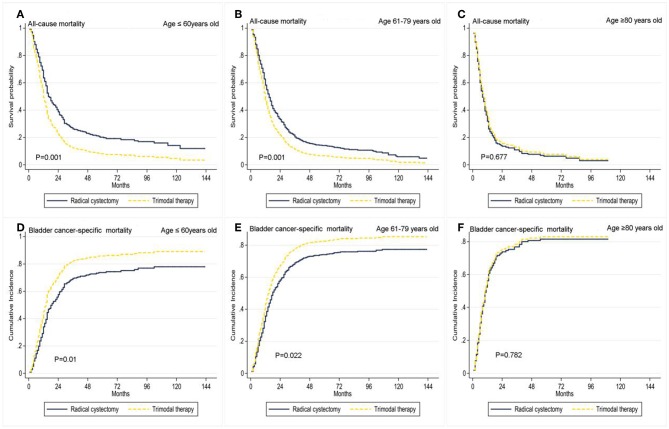
Adjusted survival curves for all-cause mortality and bladder cancer-specific mortality by radical cystectomy (**A**, age ≤ 60 years old; **B**, age 61–79 years old; **C**, age ≥ 80 years old) and trimodal therapy (**D**, age ≤ 60 years old; **E**, age 61–79 years old; **F**, age ≥ 80 years old) after weighting in age subgroups.

We further analyzed age by subgroup. Among patients aged ≤ 60 years and 61–79 years, TMT was associated with higher ACM (HR 1.57, 95% CI: 1.21–2.05, *P* = 0.001; HR 1.39, 95% CI: 1.14–1.69, *P* = 0.001, Table 4) and higher BCSM (HR 1.46, 95% CI: 1.10–1.95, *P* = 0.01; HR 1.29, 95% CI: 1.37–1.61, *P* = 0.022) (Figure 3), compared to RC. However, among patients aged ≥80 years, there was no difference in prognosis between the TMT and RC groups (*P* > 0.05).

**Table 4:** Proportional hazards regression model for all-cause mortality and bladder cancer-specific mortality according to treatment type, stratified by age.

**Covariate**	**Radical cystectomy vs. trimodal therapy**			
	**Bladder cancer-specific mortality**		**All-cause mortality**	
	**HR (95% CI)**	***P*-value**	**HR (95% CI)**	***P*-value**
Age ≤60 years	1.46 (1.10–1.95)	0.01	1.57 (1.21–2.05)	0.001
Age 61–79 years	1.29 (1.37–1.61)	0.022	1.39 (1.14–1.69)	0.001
Age ≥80 years	1.50 (0.74–1.48)	0.782	0.93 (0.65–1.33)	0.677

*Radical cystectomy as a 1 (reference) compared to trimodal therapy*.

## Discussion

NOCBCa is a heterogeneous disease. Although there are many treatments for these patients, including chemotherapy and immunotherapy, the prognosis of NOCBCa remains poor (3–5). RC has shown good survival benefits for NOCBCa (6–8), but this treatment option may not be the best choice because some patients have reached an advanced stage at the time of diagnosis or wish to retain the bladder. In recent years, TMT has been increasingly used for MIBC and has been seen as a good alternative treatment for some patients (9, 11, 13). However, the survival of TMT for NOCBCa remains clear. To provide clear clinical guidance to clinicians and patients, it is important to choose the right treatment approach for each patient.

Our research has some important findings. Among patients with NOCBCa, TMT was more commonly used than RC in patients with T1-2, N0-1, or M1, whereas RC treatment was more commonly used in patients with T3-4, N2-3, or M0. ACM was significantly decreased in patients who underwent RC instead of TMT. Our results are consistent with recent population-based studies showing that overall mortality is lower in patients who receive RC vs. TMT (9, 14). In addition, we found that patients who underwent RC had lower BCSM than patients who received TMT. Compared to TMT, the 1-, 3-, and 5-year ACM and BCSM of patients who underwent RC were lower.

Because patients with stage IV BC represent a heterogeneous population, we performed propensity matching to reduce selection bias. To determine which patients are best suited for which type of local treatment, we performed subgroup analysis for age. These analyses revealed a better prognosis for RC vs. TMT among patients aged ≤ 60 years and 61–79 years, with no statistical difference between TMT and RC in the age ≥80 years group. This result indicates that RC is still an effective treatment in younger patients; however, TMT can be selected to improve QoL and maintain bladder function in the elderly population (10, 15).

In high-risk MIBC populations, especially in patients with high surgical risk and want to preserve the bladder, treatments that preserve the bladder have been studied as an alternative treatment for RC (9, 11, 16). It is currently believed that in the treatment of bladder retention, TMT consisting of the largest TURBT followed by radiation therapy and chemotherapy produces the best oncologic effect (10, 15). In recent years, the choice of patients with TMT has gradually increased, and a large proportion of these patients are not suitable for RC due to age, disease severity and comorbidities, so this may explain the low survival rate of patients with TMT. However, our data suggest that TMT can still be used for alternative treatment for patients ≥80 years of age. Moreover, the success of TMT requires not only professional oncology and radiotherapy experts, but also a urology oncologist who is experienced in the bladder, who can safely perform a rescue cystectomy. Therefore, patients who are eligible for TMT should be offered an opportunity to discuss all alternative treatments prior to treatment selection.

Our research has some limitations. First, the study was retrospective and had inherent selection bias, although we tried to control potential bias by using propensity score matching. Second, we provide population-based TMT and RC assessments. Because the SEER database lacks specific details about the dose or period of chemotherapy and the dose of radiation, we did not evaluate which specific type of TMT was used. In addition, the SEER database lacks toxicity data and assessments of complications and QoL. Therefore, further prospective studies are needed to determine the long-term outcomes of these treatments.

## Conclusion

In summary, we found that RC is associated with significantly decreased ACM and BCSM when compared to TMT. However, the survival benefit and the risk of RC should be weighed, especially in elderly patients, and our results suggest that there may be no difference in prognosis between RC and TMT in patients with age ≥80 years. These results are preliminary and emphasize the need for randomized controlled trials to compare TMT with RC.

## Data Availability Statement

Publicly available datasets were analyzed in this study. This data can be found here: https://seer.cancer.gov.

## Author Contributions

YG: data curation. WZ and JZ: formal analysis. PW and XY: funding acquisition, supervision, and writing—review & editing. SM and RW: investigation. AZ and LW: methodology. YY and XY: project administration. XY: resources. AZ and ZZ: software. XJ and YW: validation. YG and XJ: visualization and writing—original draft.

### Conflict of Interest

The authors declare that the research was conducted in the absence of any commercial or financial relationships that could be construed as a potential conflict of interest.
